# Association analysis of the sorting nexin 29 (SNX29) gene copy number variations with growth traits in Diannan small-ear (DSE) pigs

**DOI:** 10.1080/10495398.2024.2309956

**Published:** 2024-02-05

**Authors:** Long Yang, Xiaoding Lin, Yuhan Chen, Peiya Peng, Qun Lan, Heng Zhao, Hongjiang Wei, Yulong Yin, Mei Liu

**Affiliations:** a College of Animal Science and Technology, Hunan Agricultural University, Changsha, China; b Yunnan Province Key Laboratory for Porcine Gene Editing and Xenotransplantation, Yunnan Agricultural University, Kunming, China

**Keywords:** *SNX29*, copy number variation, growth traits, associations, Diannan small-ear pig

## Abstract

*SNX29* is a potential functional gene associated with meat production traits. Previous studies have shown that *SNX29* copy number variation (CNV) could be implicated with phenotype in goats. However, in Diannan small-ear (DSE) pigs, the genetic impact of *SNX29* CNV on growth traits remains unclear. Therefore, this study investigated the associations between *SNX29* CNVs (CNV10810 and CNV10811) and growth traits in 415 DSE pigs. The results revealed that the CNV10810 mutation was significantly associated with backfat thickness in DSE pigs at 12 and 15 months old (*P* < 0.05), while the CNV10811 mutation had significant effects on various growth traits at 6 and 12 months old, particularly for body weight, body height, back height and backfat thickness (*P* < 0.05 or *P* < 0.001). In conclusion, our results confirm that *SNX29* CNV plays a role in regulating growth and development in pigs, thus suggesting its potential application for pig breeding programmes.

## Introduction

Pork is one of the most essential meat sources for humans.^1^ Growth performance and fat deposition have been extensively studied in animal genetics and breeding in pigs as important economic traits in domestic pigs.^2^ Researchers genetically develop pigs to improve the efficiency of pork production and meat flavor to meet the requirements of consumers.^3^ In China, the demand for pork products has been continuously increasing due to the steady population growth and the rapid development of society.^4^ As a local pig breed in China, DSE pigs have better meat quality and higher-fat deposition than western pigs. The DSE pig (Supplementary Figure 1), which grows in southern Yunnan Province, has gradually adapted to the tropical climate of high temperature and high humidity during a long-term process of natural selection.^5^ Insufficient expression of genetic resource value in DSE pigs. Thus, due to its rarity, steadily promoting scientific protection for sustainable development is necessary.

With the improvement of sequencing technology, more and more gene copy number duplications and deletions have been discovered by researchers.^6^ In the previous studies, many molecular markers have been used to assess economic traits in animal breeding, including single-nucleotide polymorphisms (SNP), indels (Insertion/Deletion), and structural variations, such as copy number variations (CNVs), inversions, duplications and deletions.^7^^,^^8^ CNV, generally defined as a large-scale structural variation ranging from 50 bp to 5 Mb within the genome, could affect gene expression and serve as a fundamental driver of genetic diversity and phenotypic trait variability. Molecular quantitative genetics has emerged as a result of advancements in the fields of molecular genetics and molecular biology. This interdisciplinary approach combines the principles of molecular and quantitative genetics, with a specific focus on detecting and mapping Quantitative Trait Loci (QTL) or significant genes, as well as conducting studies on marker-assisted selection (MAS), marker-assisted import ,and other related areas.^9^ Notably, researchers have shown significant interest in studying important livestock traits such as meat quality, growth rate, reproduction ability ,and disease resistance. A genomic analysis based on next-generation sequencing data was conducted to identify Copy Number Variations (CNVs) segregating in an Iberian × Landrace backcross population with the aim of investigating their association with fatty acid composition and growth-related traits. Several identified CNVRs contain relevant functional genes, including *CLCA4, CYP4X1, GPAT2, MOGAT2, PLA2G2A* and *PRKG1.*^10^

The sorting connexin (SNX) family has more than 30 members in the cytoplasm and membrane binding sites that may potentially bind to the PX domain through their lipids or interactions between proteins and membrane-associated protein complexes. SNX family is characterized by the involvement of PX domains in cell-related functions, such as cell signaling and vesicular trafficking. *SNX29* gene belongs to the SNXs family. The main function of the *SNX29* gene is to bind to microtubule motor protein activity and phosphatidylinositol, which plays a vital role in substance transport and lipid metabolism. In addition, the *SNX29* gene is also a candidate gene for carcass and back fat traits. In Yorkshire pigs, a genome-wide association analysis (GWAS) has identified that SNPs in the intron of *SNX29* were associated with intramscular fat (IMF).^11^ However, reports on this gene in DSE pigs are still lacking.

This study aimed to elucidate the possible effects of *SNX29* CNVs on growth traits and backfat traits of DSE pigs at different developmental stages. To this end, eight phenotypic traits, including body weight (BW), body height (BOH), body length (BL), back height (BAH), chest circumference (CC), cannon bone circumference (CBC), abdominal circumference (AC) and backfat thickness (BF) were firstly measured from 415 DSE pigs. Then, the association analysis of these phenotypic traits with the *SNX29* CNVs was performed. The results provide a basis for molecular marker-assisted breeding and genetic improvement of Chinese local breeding pigs.

## Materials and methods

### Ethics statement

The experiments carried out in this study were approved by the International Animal Protection and Utilization Committee of Hunan Agricultural University (protocol number: CACAHU 20210701) and complied with local laws and policies on animal welfare.

### Animals and phenotypic recording

Among the subjects of this study, all DSE pigs (205 males and 210 females) were derived from the Sipsongpanna breeding farm (a national core DSE pig conservation farm in China). A total of 415 individuals, bred in the same nutritional and management conditions, with different age stages (3, 6, 9, 12 and 15 months old) were selected for measuring the growth and obesity-related traits. The recorded phenotypic values were BW, BOH, BAH, CC, BL, CBC, AC and BF. Six body measurement traits (BOH, BAH, CC, BL, CBC and AC) were measured by tape or meter ruler. The A-mode ultrasonography (Renco lean meter®, Minneapolis, MN, USA) was used to determine BF at 3rd and 4th last ribs (near the dorsal midline at 5 centimeters). BW was recorded by weighing scale. Ear tissues were collected from all pigs and stored at −80 °C immediately after collection.

### DNA extraction and primer design

Genomic DNA was extracted from ear samples by the phenol–chloroform method,^12^ the concentration of the sample was determined by Nanodrop one spectrophotometer (Thermo Fisher Scientific, Waltham, MA, USA), and the DNA was diluted to 20 ng/µL and stored at −20 °C.

The copy number variation regions *SNX29* CNV10810 and *SNX29* CNV10811 are located at chr 3: 30607202-30609600 and chr 3: 31094802-31096800 of the reference genome sequence NC_010444.4, respectively. Primers for *SNX29* CNV analysis were designed using PrimerPremier5.0 software (Table 1 and Supplementary Figure 2). Quantitative real-time polymerase chain reaction (qPCR) was used to verify the quality of primers and the optimal temperature. Amplification curves and unchained peaks (Supplementary Figure 3) were used to determine primer specificity. Cycle threshold (Ct) values were then used for qPCR analysis.

**Table 1.** Details of primers used for pig *SNX29* CNV detection in this study.

Gene	Pairs Sequence (5′-3′)	qPCR Amplification Length (bp)	Tm (°C)
*SNX29*-CNV 10810	F:CTCTGAGCGTCCCTCTTCT	178	60
	R:GCTTCTACCTGCCTCTTCA		
*SNX29*-CNV 10811	F:ATCATGGCTCAGTGGAAAT	105	60
	R:AGTGCGTATGGAACCTACA		
*GCG*	F:GAATCAACACCATCGGTCAAAT	147	60
	R:CTCCACCCATAGAATGCCCAGT		

Note: F: forward primer; R: reverse primer.

### Copy number analysis of pig SNX29 gene

LightCyler® 96 quantitative polymerase chain reaction (qPCR) system with SYBR green dye (Roche, Basel, Switzerland) was used in this study. The GCG gene was selected as the reference gene. The total volume of the reaction mixture was 10 μL, and the 10 μL reaction system contained 5 μL SYBR®Premix Ex Taq II, 1.5 μL genomic DNA, 1 μL each of the forward and reverse primers (Table S1), and 1.5 μL ddH_2_O. The experimental procedure was as follows: 95 ◦C for 45 s, 45 cycles for 15 s, 95 ◦C for 5 s, and 60 ◦C for 40 s. All experiments were performed in triplicate, and the results were expressed as the mean ± standard error (SE).

### Statistical analyses

The copy number of the *SNX29* gene in this study was calculated by 2 × 2^−ΔΔCt^(ΔCt = Ct*_SNX29_*_-CNV_–Ct*_GCG_*; ΔΔCt= ΔCt_test sample_–ΔCt_control sample_).^13^ According to formula 2 × 2^−ΔΔCt^, the *SNX29* CNV was divided into three types, including loss type(copy number <2), normal type(copy number =2) and gain type (copy number >2). The association between pig *SNX29* gene CNV types and growth traits in different age stages was analyzed using one-way analysis of variance (ANOVA) method in SPSS software using the following reduced model: Y_i_ = *μ* + CNV_i_ + e. Y_i_ was the observation of the phenotypic traits, *μ* was the overall mean of each trait, and e_i_ was the random residual error. CNV_i_ was the effect due to the CNV type. Differences were considered significant if the *P*-value was <0.05 after multiple comparison corrections. The sex effect was not significant, so the sex effect was not added to the reduced model.

## Results

### Distribution of SNX29 CNVs in DSE pigs

**Figure 1.**
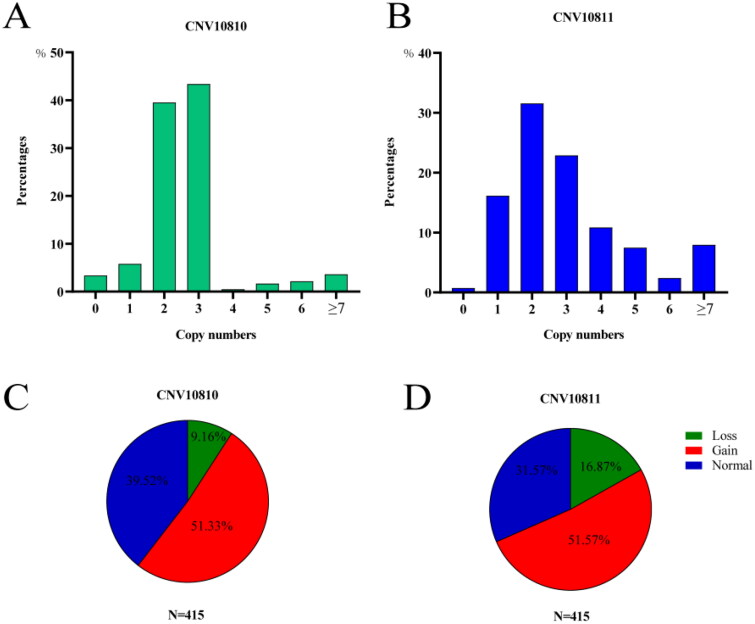
Distribution of different *SNX29* CNV types in CNV10810 and CNV10811 in the 415 DSE pigs.

Previous research has demonstrated the presence of two copy number variation (CNV) types, CNV10810 and CNV10811, in the *SNX29* gene of Duroc pigs.^14^ The objective of this study was to investigate whether *SNX29* CNV also exists in DSE pigs. Our findings revealed that gain-type variants were detected at frequencies of 51.33% for CNV10810 and 51.57% for CNV10811 within the total subjects (Fig. 1). Additionally, loss-type variants accounted for 9.16% and 16.87% in CNV10810 and CNV10811, respectively, while normal-type variants constituted 39.52% and 31.57%, respectively (Fig. 1). These results confirmed the existence of *SNX29* CNVs in DSE pigs, thereby highlighting the potential significance of investigating associations between different *SNX29* CNV types and growth traits.

### Association analysis between SNX29 CNV and growth traits at different ages of DSE pigs

To investigate the association between the *SNX29* CNV types and the growth traits of the DSE pigs, eight body size traits of DSE pigs with five age stages (3, 6, 9, 12 and 15 months) were measured, while the *SNX29* CNV types were detected in 415 individuals.

For CNV10810, the loss-type exhibited a significant phenotypic advantage over the gain-type and the normal-type, with greater backfat thickness in 15-month-old and 12-month-old DSE pigs (*P* < 0.05). Interestingly, in 15-month-old DSE pigs, the body length of individuals with the loss-type was significantly lower than that of the gain and normal types (*P* < 0.05) (Table 2). In DSE pigs aged 3rd, 6th and 9th months, there were no significant differences (Supplementary Table S1).

**Table 2.** Association analysis of *SNX29* CNV10810 and growth traits in DSE pigs.

Age	Growth Trait	CNV Type (Mean ± SE)			*P*-Value
		Normal(*n* = 164)	Loss(*n* = 38)	Gain(*n* = 213)	
12th month	BW(kg)	34.56 ± 1.44	38.53 ± 2.14	34.69 ± 0.98	0.16
	BOH(cm)	46.74 ± 0.63	48.21 ± 1.20	46.27 ± 0.62	0.25
	BL(cm)	83.54 ± 1.31	80.70 ± 1.78	82.62 ± 1.04	0.14
	BAH(cm)	78.18 ± 1.32	83.21 ± 2.20	78.76 ± 1.03	0.07
	CC(cm)	45.49 ± 0.61	46.62 ± 1.24	46.48 ± 0.65	0.54
	CBC(cm)	92.73 ± 2.87	92.06 ± 2.47	87.48 ± 1.53	0.42
	AC(cm)	12.28 ± 0.19	12.41 ± 0.19	12.13 ± 0.16	0.59
	BF(mm)	22.85 ± 0.84 ^b^	25.26 ± 1.00 ^a^	23.07 ± 0.64 ^a,b^	0.04*
15th month	BW(kg)	43.05 ± 1.00	44.48 ± 2.05	44.02 ± 0.98	0.73
	BOH(cm)	48.56 ± 0.56	49.06 ± 0.75	49.81 ± 0.50	0.24
	BL(cm)	90.16 ± 0.86 ^ab^	85.40 ± 4.28 ^b^	91.59 ± 0.75 ^a^	0.05*
	BAH(cm)	86.43 ± 0.93	83.36 ± 4.52	87.13 ± 0.99	0.69
	CC(cm)	47.04 ± 0.58	47.77 ± 0.71	48.39 ± 0.49	0.19
	CBC(cm)	93.55 ± 2.39	96.85 ± 1.97	96.47 ± 0.99	0.37
	AC(cm)	12.66 ± 0.10	12.71 ± 0.18	12.76 ± 0.12	0.83
	BF(mm)	22.98 ± 0.93 ^b^	25.37 ± 1.04 ^a^	23.04 ± 0.82 ^ab^	0.01*

Notes: Values with different letters (a,b) within the same row represent significant differences among the three groups. (*P < 0.05).

For CNV10811, as shown in Table 3, among the 6th, 9th and 12th-month-old DSE pig populations, individuals with gain type had better growth performance, such as BW and BOH (*P* < 0.05) and BAH (*P* < 0.01) in the 12-month-old individuals, BW, BF (*P* < 0.001), and BAH (*P* < 0.01) in 6-month-old individuals. However, the individuals with CNV loss type had significantly higher CC than those with CNV gain and normal types (*P* < 0.001). There were no significant differences both in 3rd and 15th-month-old DSE pigs (Supplementary Table S2).

**Table 3.** Association analysis of *SNX29* CNV10811 and growth traits in DSE pigs.

Age	Growth Trait	CNV Type (Mean ± SE)			*P*-Value
		Normal(*n* = 131)	Loss(*n* = 70)	Gain(*n* = 214)	
6th month	BW(kg)	18.54 ± 0.64^b^	22.86 ± 0.84^a^	23.00 ± 1.81 ^a^	0.00**
	BOH(cm)	36.68 ± 0.56	39.22 ± 1.63	36.63 ± 0.70	0.16
	BL(cm)	68.16 ± 1.13	71.49 ± 0.80	68.80 ± 1.29	0.27
	BAH(cm)	60.78 ± 0.73^b^	64.79 ± 0.82^a^	64.21 ± 1.45 ^a^	0.00**
	CC(cm)	35.70 ± 0.52^b^	40.08 ± 0.87 ^a^	33.03 ± 2.09 ^b^	0.00**
	CBC(cm)	68.57 ± 0.96	71.56 ± 1.12	70.58 ± 1.85	0.14
	AC(cm)	10.79 ± 0.10	10.74 ± 0.11	10.73 ± 0.19	0.92
	BF(mm)	17.63 ± 0.43 ^b^	18.33 ± 0.47 ^b^	21.00 ± 0.77 ^a^	0.01**
9th month	BW(kg)	24.63 ± 1.07	25.95 ± 1.58	25.65 ± 1.07	0.65
	BOH(cm)	41.21 ± 0.60	41.69 ± 0.86	41.43 ± 0.81	0.90
	BL(cm)	72.19 ± 0.92	73.54 ± 1.42	73.23 ± 1.88	0.70
	BAH(cm)	66.22 ± 0.99	67.31 ± 1.27	69.25 ± 1.38	0.33
	CC(cm)	39.69 ± 0.60	39.76 ± 0.87	40.34 ± 0.97	0.88
	CBC(cm)	74.39 ± 1.03	76.91 ± 1.49	77.56 ± 1.46	0.21
	AC(cm)	10.49 ± 0.10 ^b^	11.01 ± 0.19 ^a^	10.78 ± 0.21^a,b^	0.03*
	BF(mm)	20.21 ± 0.37	20.75 ± 0.84	21.95 ± 1.19	0.23
12th month	BW(kg)	34.36 ± 1.23^a,b^	30.94 ± 2.11 ^b^	37.02 ± 1.09 ^a^	0.04*
	BOH(cm)	46.39 ± 0.60^a,b^	43.78 ± 1.11 ^b^	47.71 ± 0.62^a^	0.01*
	BL(cm)	83.32 ± 1.18	82.33 ± 1.75	84.22 ± 1.12	0.70
	BAH(cm)	78.36 ± 1.31^a,b^	73.83 ± 2.42^b^	81.32 ± 1.00^a^	0.01**
	CC(cm)	45.58 ± 0.64	44.04 ± 1.29	47.05 ± 0.62	0.06
	CBC(cm)	113.03 ± 2.59	83.16 ± 2.97	89.95 ± 1.41	0.47
	AC(cm)	12.33 ± 0.15	11.88 ± 0.23	12.24 ± 0.16	0.45
	BF(mm)	22.82 ± 0.61	24.72 ± 1.26	24.57 ± 0.74	0.17

Notes: Values with different letters (a,b) within the same row represent significant differences among the three groups. (* *P* < 0.05, ** *P* < 0.01).

## Discussion

In Chinese local pigs, how to elevate the performance of low growth traits are the major challenges for breeders. Growth traits such as BW, BOH, back height and backfat thickness are important to improve performance defects and economic benefits of local pigs. Nowadays, to improve the meat production efficiency and product quality, more and more researchers pay attention to identify the useful genetic variations related with phenotypic traits in livestock.^15^^,^^16^ To our knowledge, various genetic variations have been identified as molecular markers for animal breeding. For example, previous study has shown that the *GHSR* gene SNP had positive effects on improving the performance of pig breeds.^17^ The SNP in the coding region of *KDM4D* gene was detected to be associated with testicular morphological traits in male pigs.^18^ For CNV, it could have a wider range of genetic effects than other molecular markers because it is involved in more sequence variation within the genome.^19^ In recent years, an increasing number of studies have demonstrated the impacts of CNV on growth, reproduction, and disease traits in domestic animals.^20^^,^^21^ For example, some studies have shown that *EIF4A2* CNV and *CCDC39* CNV have essential impact on body measurement traits in cattle.^22^^,^^23^ In the local Min pig, 15 CNVRs were found to be associated with pig villus growth.^20^ In the previous studies by our group, the *SNX29* CNV was found to be implicated with phenotype, such as the chest width in African goats^24^ and chest circumference and abdominal circumference in Chinese goats.^25^ In pigs, this investigation on *SNX29* CNV was the first report in the Chinese local pig population.

In this study, the results of association analysis showed that *SNX29* CNV10810 was significantly correlated with BF and BL in DSE pigs at 12- and 15-month old, while *SNX29* CNV10811 was significantly associated with BW, BOH, BAH and BF traits in DSE pigs at 6- and 12-month old. On one hand, these associations could be due to the important functions of CNV, which can alter gene expression through positional effects, fusion effects, gene blocking,and other pathways, thereby affecting phenotypic traits.^26^ On the other hand, *SNX29* gene can bind to microtubule motor protein activity and phosphatidylinositol and play an essential role in substance transport and lipid metabolism.^27^ Moreover, *SNX29* has been identified to be a candidate functional gene related to meat traits such as the slaughter traits (e.g., backfat thickness) and meat quality traits (e.g., content of intramuscular fat) in pigs,^11^ which is in accordance with the results in this study. BF is one of the most important fatness traits for pigs. Intramuscular adipose deposition is one of the key factors for meat quality in commercial pigs. In our previous report, *MCT1* and *CHCHD3* were identified as important candidate genes that were related to fat deposition.^28^ In pig breeding, abnormal body conditions not only affect the fattening and reproductive performance of pigsbut also increases the feeding cost and reduces the income.^13^ Studies have shown that sows with thick back fat have more total litters per litter than sows with thin back fat,^29^^,^^30^ but also with more stillbirths per litter,^31^ indicating the importance of appropriate backfat thickness in sows. Comprehensively, in this study, the loss type of *SNX29* CNV10810 and the gain type of *SNX29* CNV10811 should have better advantages.

As a method of modern molecular breeding, molecular marker-assisted selection (MAS) has the advantages of high efficiency, stable and reliable results,and good repeatability.^32^ Meanwhile, as typical types of molecular markers, SNPs and InDels have been widely used in animal genetics and breeding, whereas CNV is rarely used in breeding and has attracted attention in recent years due to its greater effects on sequence variations.^33^^,^^34^ In this study, association results indicated that the two CNVs in *SNX29* could be used as key molecular markers for improving meat quality and/or meat production of DSE pigs.

However, the results in this study need to be verified in more samples and other breeds. Moreover, genomic CNV can affect the phenotype of an organism in several ways, including gene dosage alteration and gene expression regulation, and is heritable, relatively stable,and highly heterogeneous. It has been shown that duplications and deletions of cis-regulatory elements can also significantly affect the phenotype, especially when such CNVs affect developmentally relevant genes.^35^ Thus, the molecular regulation mechanism of *SNX29* CNV’s impacts on pig growth traits needs to be further studied.

In conclusions, this study described the distribution of *SNX29* gene CNVs in DSE pigs for the first time. The association analysis between *SNX29* CNVs (CNV10810 and CNV10811) and growth traits revealed that CNV10810 was significantly associated with BF of DSE pigs at 12 and 15 months old, while CNV10811 was significantly correlated with growth traits such as BF and BH at 6 and 12 months old. These results indicated that *SNX29* CNVs may be related to the growth and development of DSE pigs and these two CNVs can be used as the molecular markers for early MAS breeding in DSE pigs.

## Supplementary Material

Supplemental Material
