# Supplementary material for: Association analysis of the sorting nexin 29 (SNX29) gene copy number variations with growth traits in Diannan small-ear (DSE) pigs
Source: Anim Biotechnol. 2024 Feb 5;35(1):2309956. doi: 10.1080/10495398.2024.2309956 (PMC12674349; doi:10.1080/10495398.2024.2309956)
Supplement: Supplemental Material [file LABT_A_2309956_SM5333.docx]

**Supplementary materials**

Supplementary Table 1 . Association analysis of SNX29 CNV10810 and growth traits in all DSE pigs.

| Age | Growth Trait | CNV Type (Mean ± SE) | | | *P*-Value |
| --- | --- | --- | --- | --- | --- |
|  |  | Normal(n=164) | Loss(n=38) | Gain(n=213) |  |
| 3rd month | BW（kg） | 8.96±0.31 | 10.33±0.91 | 9.84±0.28 | 0.22 |
|  | BOH（cm） | 28.58±0.56 | 28.25±1.02 | 29.91±0.34 | 0.65 |
|  | BL（cm） | 53.26±1.06 | 56.74±1.01 | 55.05±0.79 | 0.13 |
|  | BAH（cm） | 48.83±0.90 | 52.12±1.74 | 50.09±0.50 | 0.32 |
|  | CC（cm） | 27.45±0.72 | 28.46±1.62 | 28.71±0.39 | 0.41 |
|  | CBC（cm） | 51.23±0.49 | 53.72±1.87 | 53.46±1.07 | 0.11 |
|  | AC（cm） | 9.24±0.17 | 9.34±0.25 | 13.37±2.86 | 0.35 |
| 6th month | BW（kg） | 21.33±0.62 | 20.87±0.84 | 19.39±1.03 | 0.65 |
|  | BOH（cm） | 38.15±0.89 | 38.23±1.63 | 36.41±0.71 | 0.34 |
|  | BL（cm） | 82.70±1.25 | 70.59±0.80 | 67.77±1.22 | 0.22 |
|  | BAH（cm） | 63.38±0.57 | 64.63±0.92 | 61.29±1.08 | 0.68 |
|  | CC（cm） | 37.45±0.76 | 41.58±0.89 | 35.51±0.78 | 0.33 |
|  | CBC（cm） | 71.19±0.79 | 70.54±1.14 | 67.61±1.19 | 0.65 |
|  | AC（cm） | 10.84±0.08 | 10.71±0.12 | 10.66±0.12 | 0.34 |
|  | BF（mm） | 18.61±0.39 | 18.32±0.48 | 18.13±0.61 | 0.38 |
| 9th month | BW（kg） | 24.21±1.26 | 25.58±4.93 | 25.28±1.00 | 0.79 |
|  | BOH（cm） | 41.23±0.71 | 42.00±1.50 | 41.43±0.57 | 0.94 |
|  | BL（cm） | 71.23±1.04 | 75.00±10.80 | 73.80±0.87 | 0.17 |
|  | BAH（cm） | 66.03±1.19 | 70.10±7.90 | 67.53±0.83 | 0.44 |
|  | CC（cm） | 39.36±0.69 | 40.75±2.25 | 40.13±0.6 | 0.65 |
|  | CBC（cm） | 74.54±1.24 | 77.40±7.10 | 76.15±0.95 | 0.54 |
|  | AC（cm） | 10.58±0.12 | 10.70±0.30 | 10.72±0.12 | 0.75 |
|  | BF（mm） | 20.08±0.53 | 19.75±1.25 | 21.10±0.48 | 0.33 |
| 12th month | BW（kg） | 34.56±1.44 | 38.53±2.14 | 34.69±0.98 | 0.16 |
|  | BOH（cm） | 46.74±0.63 | 48.21±1.20 | 46.27±0.62 | 0.25 |
|  | BL（cm） | 83.54±1.31 | 80.70±1.78 | 82.62±1.04 | 0.14 |
|  | BAH（cm） | 78.18±1.32 | 83.21±2.20 | 78.76±1.03 | 0.07 |
|  | CC（cm） | 45.49±0.61 | 46.62±1.24 | 46.48±0.65 | 0.54 |
|  | CBC（cm） | 92.73±2.87 | 92.06±2.47 | 87.48±1.53 | 0.42 |
|  | AC（cm） | 12.28±0.19 | 12.41±0.19 | 12.13±0.16 | 0.59 |
|  | BF（mm） | 22.85^b^±0.84 | 25.26^a^±1.00 | 23.07^ab^±0.64 | 0.04^*^ |
| 15th month | BW（kg） | 43.05±1.00 | 44.48±2.05 | 44.02±0.98 | 0.73 |
|  | BOH（cm） | 48.56±0.56 | 49.06±0.75 | 49.81±0.50 | 0.24 |
|  | BL（cm） | 90.16±0.86^ab^ | 85.40±4.28^b^ | 91.59±0.75^a^ | 0.05^*^ |
|  | BAH（cm） | 86.43±0.93 | 83.36±4.52 | 87.13±0.99 | 0.69 |
|  | CC（cm） | 47.04±0.58 | 47.77±0.71 | 48.39±0.49 | 0.19 |
|  | CBC（cm） | 93.55±2.39 | 96.85±1.97 | 96.47±0.99 | 0.37 |
|  | AC（cm） | 12.66±0.10 | 12.71±0.18 | 12.76±0.12 | 0.83 |
|  | BF（mm） | 22.98±0.93^b^ | 25.37±1.04^a^ | 23.04±0.82^ab^ | 0.01^*^ |

Notes: Values with different letters (a,b) within the same row represent significant differences among the three groups. (* *P*< 0.05, ** *P* < 0.01).

Supplementary Table 2. Association analysis of SNX29 CNV10811 and growth traits in all DSE pigs

| Age | Growth Trait | CNV Type (Mean ± SE) | | | *P*-Value |
| --- | --- | --- | --- | --- | --- |
|  |  | Normal(n=131) | Loss(n=70) | Gain(n=214) |  |
| 3rd month | BW（kg） | 10.18±0.33 | 10.15±0.90 | 9.24±0.36 | 0.15 |
|  | BOH（cm） | 30.42±0.42 | 29.24±1.04 | 29.06±0.43 | 0.08 |
|  | BL（cm） | 55.44±1.06 | 56.94±2.00 | 53.99±0.96 | 0.40 |
|  | BAH（cm） | 50.13±0.65 | 51.12±1.64 | 49.62±0.68 | 0.66 |
|  | CC（cm） | 29.08±0.44 | 28.36±1.52 | 27.98±0.53 | 0.30 |
|  | CBC（cm） | 54.52±0.71 | 54.82±1.88 | 51.71±1.61 | 0.26 |
|  | AC（cm） | 9.55±0.15 | 9.44±0.28 | 9.35±0.20 | 0.73 |
| 6th month | BW（kg） | 18.54±0.64^b^ | 22.86±0.84^a^ | 23.00±1.81^a^ | 0.00^**^ |
|  | BOH（cm） | 36.68±0.56 | 39.22±1.63 | 36.63±0.70 | 0.16 |
|  | BL（cm） | 68.16±1.13 | 71.49±0.80 | 68.80±1.29 | 0.27 |
|  | BAH（cm） | 60.78±0.73^b^ | 64.79±0.82^a^ | 64.21±1.45^a^ | 0.00^**^ |
|  | CC（cm） | 35.70±0.52^b^ | 40.08±0.87^a^ | 33.03±2.09^b^ | 0.00^**^ |
|  | CBC（cm） | 68.57±0.96 | 71.56±1.12 | 70.58±1.85 | 0.14 |
|  | AC（cm） | 10.79±0.10 | 10.74±0.11 | 10.73±0.19 | 0.92 |
|  | BF（mm） | 17.63±0.43^b^ | 18.33±0.47^b^ | 21.00±0.77^a^ | 0.01^**^ |
| 9th month | BW（kg） | 24.63±1.07 | 25.95±1.58 | 25.65±1.07 | 0.65 |
|  | BOH（cm） | 41.21±0.60 | 41.69±0.86 | 41.43±0.81 | 0.90 |
|  | BL（cm） | 72.19±0.92 | 73.54±1.42 | 73.23±1.88 | 0.70 |
|  | BAH（cm） | 66.22±0.99 | 67.31±1.27 | 69.25±1.38 | 0.33 |
|  | CC（cm） | 39.69±0.60 | 39.76±0.87 | 40.34±0.97 | 0.88 |
|  | CBC（cm） | 74.39±1.03 | 76.91±1.49 | 77.56±1.46 | 0.21 |
|  | AC（cm） | 10.49±0.10^b^ | 11.01±0.19^a^ | 10.78±0.21^ab^ | 0.03^*^ |
|  | BF（mm） | 20.21±0.37 | 20.75±0.84 | 21.95±1.19 | 0.23 |
| 12th month | BW（kg） | 34.36±1.23^ab^ | 30.94±2.11^b^ | 37.02±1.09^a^ | 0.04^*^ |
|  | BOH（cm） | 46.39±0.60^ab^ | 43.78±1.11^b^ | 47.71±0.62^a^ | 0.01^*^ |
|  | BL（cm） | 83.32±1.18 | 82.33±1.75 | 84.22±1.12 | 0.70 |
|  | BAH（cm） | 78.36±1.31^ab^ | 73.83±2.42^b^ | 81.32±1.00^a^ | 0.01^**^ |
|  | CC（cm） | 45.58±0.64 | 44.04±1.29 | 47.05±0.62 | 0.06 |
|  | CBC（cm） | 113.03±2.59 | 83.16±2.97 | 89.95±1.41 | 0.47 |
|  | AC（cm） | 12.33±0.15 | 11.88±0.23 | 12.24±0.16 | 0.45 |
|  | BF（mm） | 22.82±0.61 | 24.72±1.26 | 24.57±0.74 | 0.17 |
| 15th month | BW（kg） | 42.65±1.58 | 43.46±2.68 | 44.04±0.78 | 0.76 |
|  | BOH（cm） | 47.51±0.80 | 49.41±1.19 | 49.57±0.38 | 0.09 |
|  | BL（cm） | 88.36±1.22 | 85.59±7.14 | 90.96±0.63 | 0.15 |
|  | BAH（cm） | 85.45±1.57 | 88.13±2.12 | 84.89±1.60 | 0.73 |
|  | CC（cm） | 46.19±0.83 | 48.48±1.22 | 48.05±0.37 | 0.11 |
|  | CBC（cm） | 94.01±1.90 | 90.75±7.60 | 96.52±0.76 | 0.18 |
|  | AC（cm） | 12.54±0.19 | 12.73±0.19 | 12.75±0.09 | 0.57 |
|  | BF（mm） | 23.85±1.16 | 23.29±1.06 | 22.41±0.48 | 0.42 |

Notes: Values with different letters (a,b) within the same row represent significant differences among the three groups. (* *P*< 0.05, ** *P* < 0.01).


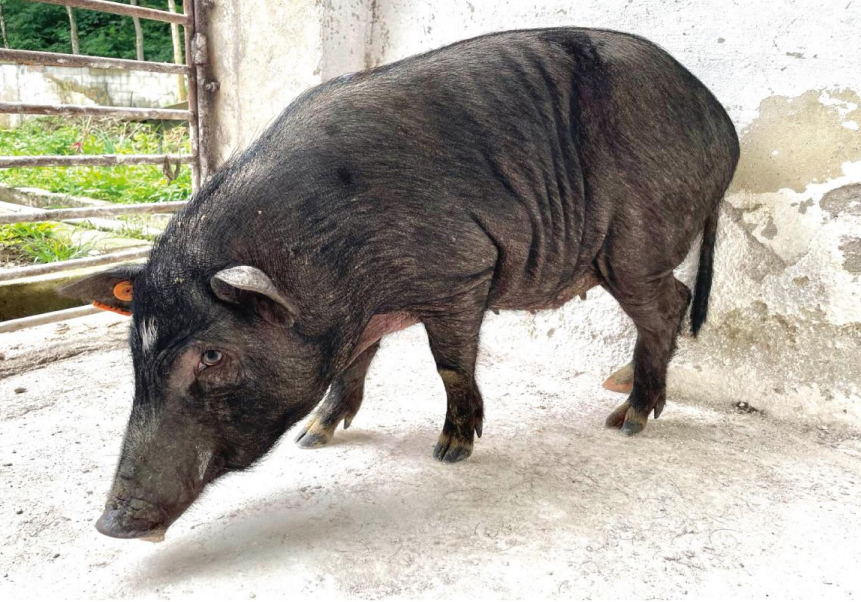


Supplementary Figure 1. Diannan small-ear (DSE) pig.


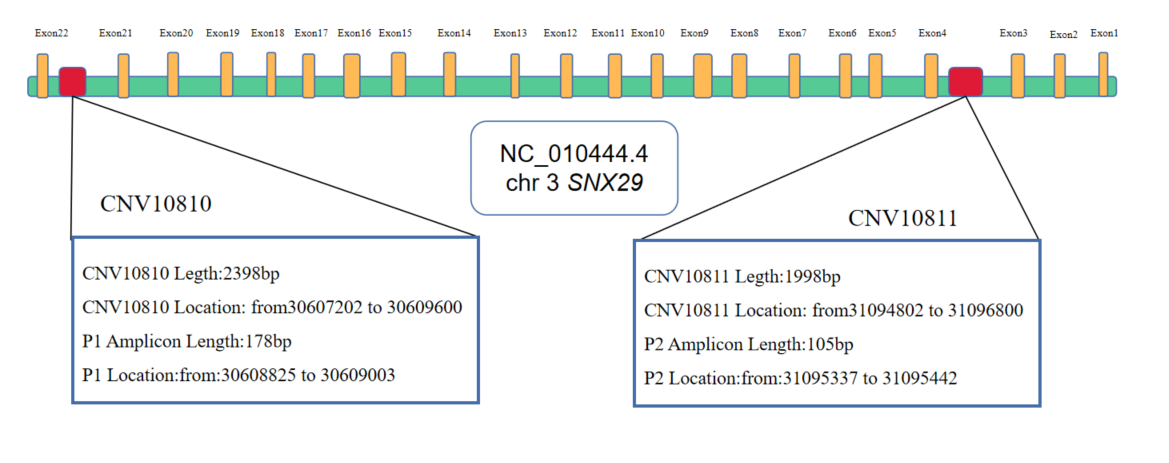


Supplementary Figure 2.Two copy number variation regions of the *SNX29* gene. Yellow boxes represent coding regions and red boxes represent CNVs.
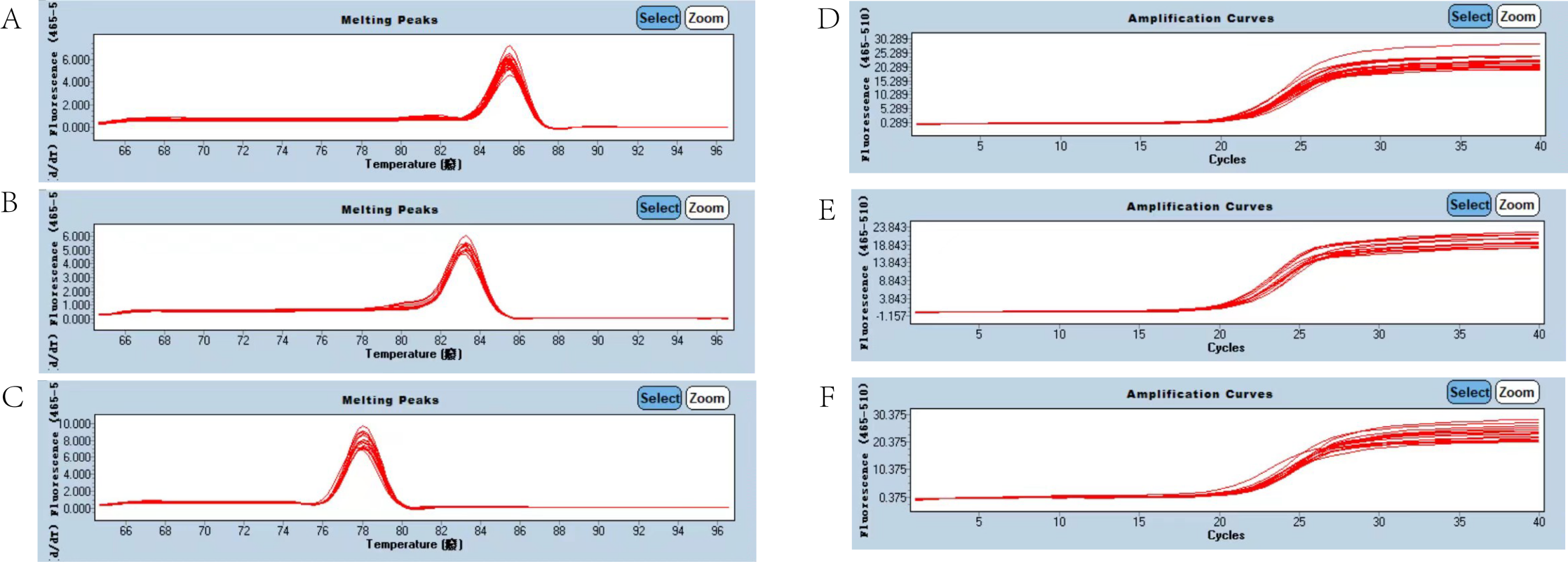


Supplementary Figure 3. Amplification curves and melt peaks of the *SNX29* CNVs and *GCG* genes.(A)The melting peak of the *GCG* gene.(B)The melting peak of *SNX29* CNV10810.(C)The melting peak of *SNX29* CNV10811.(D)Amplification curve of the *GCG* gene.(E)Amplification curve of *SNX29* CNV10810.(F)Amplification curve of *SNX29* CNV10811.
